# Relative Energy Deficiency in Sport (REDs) Indicators in Male Adolescent Endurance Athletes: A 3-Year Longitudinal Study

**DOI:** 10.3390/nu15245086

**Published:** 2023-12-12

**Authors:** Thomas Birkedal Stenqvist, Anna Katarina Melin, Monica Klungland Torstveit

**Affiliations:** 1 Department of Sport Science and Physical Education, Faculty of Health and Sport Science, University of Agder, 4630 Kristiansand, Norway; monica.k.torstveit@uia.no; 2 Department of Sport Science, Faculty of Social Sciences, Linnaeus University, 351 95 Vaxjo, Sweden; anna.melin@lnu.se

**Keywords:** adolescent athletes, bone health, energy availability, nutrition, performance, resting metabolic rate

## Abstract

Longitudinal measurements of Relative Energy Deficiency in Sport (REDs) among adolescent male elite athletes are lacking. We aimed to monitor REDs indicators and their possible impact on performance in elite high-school cross-country skiing and biathlon athletes (*n* = 13) (16.3 ± 0.4 years, 179.4 ± 7.6 cm, 63.6 ± 8.2 kg body mass (BM), and peak oxygen uptake (VO_2peak_): 61.5 ± 5.3 mL/kg BM/min) every 6 months for 3 years. Protocols included assessments of energy availability (EA), body composition and bone mineral density (BMD), resting metabolic rate (RMR), disordered eating behavior, exercise addiction, VO_2peak_, and muscle strength. Data were analyzed using a linear mixed model. At baseline, 38% had low lumbar BMD (Z-score ≤ −1), and overall, bone health increased only slightly. VO_2peak_ and muscle strength improved (*p* < 0.001), RMR decreased (*p* = 0.016), and no change was observed in EA or physiological or psychological REDs indicators. Conclusively, many of these young male athletes had poor bone health at baseline, and most either lost or did not achieve the expected pubertal bone mineral accrual, although no other indication of REDs was observed, while performance improved during the study period. Our findings highlight the importance of elite sports high schools focusing on screening for early detection of impaired bone health in male athletes.

## 1. Introduction

Relative Energy Deficiency in Sport (REDs), triggered by problematic (severe and/or prolonged) low energy availability (LEA) with or without disordered eating (DE) behavior, has wide-ranging effects on physiological and psychological functioning, performance, and general health [1]. Currently, REDs are more extensively described within endurance sports at different levels in female than in male athletes, with a reported prevalence of 23–80% among female and 15–70% among male athletes representing a variety of sports [1,2]. Similar unfavorable metabolic and endocrine alterations have been observed in males as in females, which include suppression of reproductive hormones, growth impairment, poor bone health, and impaired performance [2,3].

A recent review by Gould et al. [4] highlights that LEA among adolescents is common, with the majority of research performed among adolescent females. Among male adolescent athletes, only five cross-sectional studies have investigated EA in sports such as climbing [5], cross-country running [6], soccer [7], rink hockey [8], and acrobatic gymnastics [9]. These studies have reported an EA range of 10–55 kcal/kg fat-free mass (FFM)/day, with three studies reporting a 24–47% prevalence of LEA [5,6,7]. One of the severe clinical health outcomes of problematic LEA is impaired bone health [1]. The assessment of bone mineral density (BMD) has recently been recommended in relation to REDs development in adolescent athletes [4]. Males seem to reach peak bone mass at an age of ~20 years, with an accrual of ~25% of the total adult BMD between 13 and 19 years of age [10]. Studies investigating bone health and bone mineral accrual among adolescents have shown that participation in high-impact (e.g., gymnastics) or odd-impact (e.g., football) loading sports is associated with improved bone health compared to low-impact (e.g., running) and non-impact (e.g., cycling) sports [11,12,13]. Furthermore, Barrack et al. reported a bone mineral increase at all sites in adolescent female non-runners between 13–14 and 17–18 years of age, while no increase was noted in aged-matched distance runners [14]. Other recommended REDs indicators to screen for in adolescent athletes include, but are not limited to, disordered eating (DE) behavior and a drive for thinness [1]. Many adolescents undergo several bodily changes, as well as experiencing body pressure via social media. These factors make them extra vulnerable and exposed to challenges related to self-esteem and body image, which in turn can increase the risk of developing a difficult relationship with training, food, body weight, and shape. Athletes who are still growing and, at the same time, have a high training volume have an additional need for the right diet [15].

To prevent the occurrence and progression of REDs, the early identification of athletes at risk, such as adolescent endurance athletes, is critical [16]. Furthermore, to encourage early identification and management of REDs signs or indicators and prevent the development of more serious outcomes such as osteoporosis or eating disorders, both subjective and objective assessments at different time periods throughout a season are recommended [16].

To our knowledge, no studies have investigated REDs from a longitudinal perspective among male adolescent elite endurance athletes. The aim of this study was to monitor indicators of REDs in male adolescent endurance athletes attending Norwegian elite sports high schools over a 3-year period, focusing on health factors such as bone development, energy metabolism, and behaviors regarding food, body weight, and shape, as well as performance variables. 

## 2. Materials and Methods

### 2.1. Study Design and Participants

The duration of this longitudinal study was planned to be three academic years, with testing conducted at six time points (T_1_–T_6_, every 6 months). However, due to the coronavirus disease pandemic, T_6_ was cancelled, and testing was performed at 5 time points over 2.5 years. The study protocol was approved by the University Faculty Ethics Committee and the Norwegian Centre for Research Data (no. 54496/3/STM) on the 16 August 2017. All testing procedures complied with the principles of the Declaration of Helsinki, and all athletes signed informed consent prior to participation.

**Figure 1:**
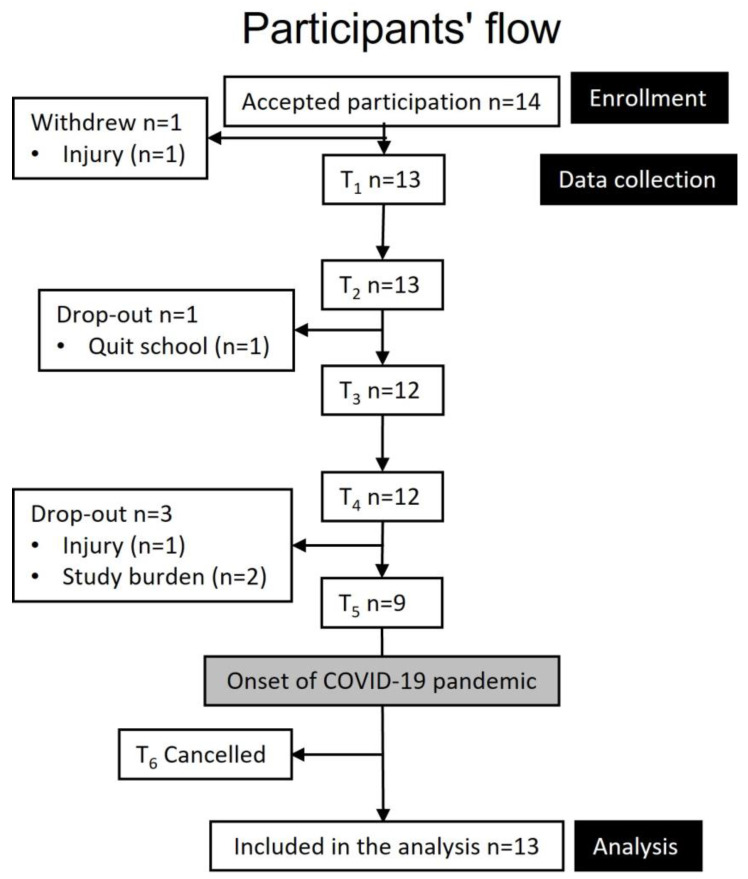
Athletes’ flow throughout the study.

Three Norwegian elite sports high schools within 200 km from the sport science laboratory on campus received information about the study, and an “on-site” presentation was provided for first-year male endurance athletes selected by their coaches based on performance ranks. The schools were organized similarly to boarding schools (athletes living together on campus) and had an employed coach responsible for the athletes’ training, both group-wise and individually. The baseline inclusion criteria were first-year students at an elite sports high school who were competing in an endurance sport at a regional or national level (Tier 3) [17] and absence of injury. A total of 14 cross-country skiing and biathlon athletes accepted participation. There was a 31% attrition from baseline to T_5_ (2.5 years later), leaving nine athletes to complete the study, as shown in Figure 1. The dropouts were due to injuries, quitting school, or the overall burden of participating while simultaneously maintaining daily training, schoolwork, competitions, and regular participation at training camps.

All athletes received information about the study and test procedures and signed an informed consent agreement. 

### 2.2. Study Protocol

Five days before testing, the athletes were instructed to log their training using a heart rate (HR) monitor. Athletes (*n* = 10) arriving from schools far from campus (>150 km) stayed overnight at the sport science laboratory before undergoing testing the next morning. Accommodation was within 50 m from the test facilities. The day before testing, an assessment of energy intake (EI) was performed. After an overnight sleep, between 6 a.m. and 9 a.m., measurements of RMR, body composition, and bone health were performed in a fasting state. Subsequently, athletes were served an ad libitum breakfast and asked to answer questionnaires assessing psychological parameters. Performance tests were performed between 11 a.m. and 2 p.m. and included assessments of peak oxygen uptake ($\dot{V}$O_2peak_), muscle power, and muscle strength. 

### 2.3. Athlete Characteristics

Height was measured without shoes to the nearest 0.1 cm using a wall-mounted stadiometer (Seca Optima, Seca, Birmingham, UK), and body mass was measured in underwear to the nearest 0.01 kg using an electronic weighing scale (Seca 861, Birmingham, UK). Body mass index (BMI) was calculated as body mass in kilograms divided by height in square meters (kg/m^2^). Body fat <5% was defined as unhealthy low levels [18], while underweight was classified as either grade 1 (<10th percentile of normal BMI) or grade 2 (<3rd percentile of normal BMI), as defined by the Norwegian growth charts for children and adolescents [19]. 

### 2.4. Resting Metabolic Rate

RMR was measured by indirect calorimetry using a canopy hood (Oxycon Pro, Jager, Hanover, Germany), and the systems were calibrated according to the laboratory standards and best-practice protocols [20]. Athletes rested for 15 min before undergoing the measurements in a supine position. Oxygen consumption (VO_2_) and carbon dioxide production (VCO_2_) were assessed over a 30 min period, with no sleeping allowed. The last 20 min of measurement were used to calculate RMR using the Weir equation (3.9 × liters oxygen consumed + 1.1 × liters carbon dioxide produced). Testing was approved with a coefficient of variation of <10% [20]. The predicted RMR was calculated using the Cunningham equation (500 + 22 × LBM (in kilograms)) and the RMR_ratio_ was calculated as measured RMR (kcal) divided by predicted RMR (kcal). 

### 2.5. Body Composition and Bone Health

Body composition, BMD, and bone mineral content (BMC) were assessed using dual-energy X-ray absorptiometry (DXA) (GE-Lunar Prodigy, Madison, WI, USA, and Encore software version 15.2, with the combined Lunar/NHANES database). Body composition was assessed according to the best-practice protocol described by Nana et al. [21], which included the assessment of hydration status via urine-specific gravity testing (USG) using a digital refractometer (Atago UG-α, Cat.No. 3464, Atago U.S.A. Inc., Bellevue, WA, USA). BMD (g/cm^2^) and BMC (g) were assessed in the lumbar spine (L1–L4), femoral neck, total hip, and total body less head (TBLH). Athletes were considered to have poor bone health if their BMD Z-score in either the TBLH or lumbar spine was ≤−1.0 [1].

### 2.6. Psychological Parameters

After the DXA measurements, three six-item short questionnaires were distributed to assess psychological parameters, including DE behavior, drive for leanness, and exercise addiction. These instruments were the Brief Eating Disorder in Athletes Questionnaire (BEDA-Q) [22], the Drive for Leanness Scale (DLS) [23], and the Exercise Addiction Inventory for Youth (EAI-Y) questionnaire [24]. 

### 2.7. Energy Intake, Exercise Energy Expenditure and Energy Availability

EI was assessed using a semi-structured dietary interview, collecting information on the athletes’ typical food patterns during the last four days [25]. The research members performing the interviews were trained in advance following an interview protocol and were equipped with images of food portions designed to aid in estimating portion sizes. The Google search engine was used to help the interviewers and athletes identify specific products. After the interview, data were logged using the Dietist Net (Dietist Net, Kost och Näringsdata, Bromma, Sweden) with access to the Norwegian food table and an open Norwegian nutritional information database. Exercise energy expenditure (EEE) was calculated using an HR monitor during all training sessions (Polar M400, Polar Electro, Kempele, Finland) four days before testing. Data were recorded as 5-second epochs during each training session. EEE (kcal/kg/min) was calculated using the following formula: EEE = ([5.95 × HRaS] + [0.23 × age] + (84 × 1) − 134)/4186.8, where HRaS was HR above sleeping HR (beats/min). Sleeping HR was derived from a supine resting measurement during RMR assessment and was calculated as 0.83 × supine HR [26]. EA was calculated as [EI (kcal) − EEE (kcal)]/fat-free mass (FFM) (kg) [1].

### 2.8. Performance

VO_2peak_ testing was performed on a treadmill (Woodway GmbH, Weil am Rhein, Germany), starting with 1 min of running at 12 km/h on a constant incline of 6° (10.5%). The speed was increased by one km/h per min until voluntary exhaustion or failure to maintain the correct position. VO_2_ and VCO_2_ were measured using Metamax 3B (Cortex Biophysik GmbH, Leipzig, Germany) and calibrated according to standard procedures. VO_2peak_ was defined as the average of the two highest 30-s consecutive VO_2_ measurements. HR was measured continuously, and blood lactate level was measured at 1 min after completion. The objective criteria for the attainment of VO_2peak_ included plateau oxygen uptake and/or HR ≥ 95% of known HR_peak_, respiratory exchange ratio ≥1.10, and blood lactate ≥ 8.0 mmol/L. 

Leg strength and power were assessed in both legs using a 10-repetition leg extension test on a pneumatic Keiser leg extension machine (AIR300, Keiser Corporation, Fresno, CA, USA). The athletes’ seating position was adjusted, aiming at a vertical femur (equivalent to a 90° knee angle) and feet placed with heels at the bottom end of the footplate. The athletes were instructed to extend both legs with maximum effort during the entire 10-repetition test. After completion, one-repetition maximum force and power were derived from the dedicated software (Keiser A420, build 2.0.0.4, Keiser Corporation, Fresno, CA, USA).

### 2.9. Statistical Analysis

Data were analyzed using Jamovi for Windows (Jamovi Project, 2020; version 1.6.3, Sydney, Australia). Continuous data were presented as mean ± standard deviation, while questionnaire data were presented as medians with interquartile ranges (25–75). Longitudinal changes were analyzed using a linear mixed model with restricted maximum likelihood estimation, with time set as fixed effects, random intercepts, and linear slopes. The intercept represents the mean baseline value at T_1_, while the slope represents the mean increase/decrease at each time point. To interpret the magnitude of bone health changes for each athlete, an exploratory approach using the reliable change index (RCI) was used: $RCI=\frac{x^{2}-x^{1}}{SE}$ [27]. In the RCI equation, x^1^ and x^2^ represent the individual scores at two time points, while the standard error (SE) was calculated using the following formula: $SE=\sqrt{{(S}_{X}^{2}+S_{Y}^{2})(1-r_{XY})},$ where $S_{X}^{2}$ and $S_{Y}^{2}$ are the variances at the time points and r_xy_ is the test–retest reliability [28]. Changes were interpreted as statistically significant when RCI was greater than 1.96 in both directions. Values between −1.96 and 1.96 indicated no reliable change between time points [27]. The RCI was calculated at three time points (T_1_, T_3_, and T_5_), measuring the total change between T_1_–T_5_, T_1_–T_3_, and T_3_–T_5_. Statistical significance was set at *p* < 0.05.

## 3. Results

### 3.1. Athlete Characteristics

Baseline data are presented in Table 1, results from the linear mixed model are presented in Table 2, and detailed bone health development, including RCI, is presented in Table 3. All anthropometric values increased (*p* < 0.001) during the period, including BMI and FFM.

At baseline, five athletes (38%) had low lumbar spine BMD values (Z-score ≤ −1.0). None of the athletes had unhealthy low body fat levels, while three athletes (23%) were classified as grade 1 underweight at baseline. Two of the athletes increased their BMI above the underweight threshold, while one athlete stayed underweight the entire study period (grade 1). All physiological data were the within normal range (Table 1).

### 3.2. Bone Health, Energy Availability and Resting Metabolic Rate

During the longitudinal assessment, all performance variables improved, all anthropometric variables except body fat percentage increased, and relative RMR and RMR_ratio_ decreased (Table 2). In terms of the psychological variables, no changes were observed in BEDA-Q (*p* = 0.321) or DLS (*p* = 0.782) scores, while EIA-Y scores decreased during the period (Table 2). No changes were observed in EI, EEE, or EA during the period. 

The linear mixed model revealed small increases in BMD, BMC, and *Z*-scores in the lumbar spine, femur neck, total hip, and TBLH (Table 2). Out of the five athletes with low BMD at baseline, one exhibited improvement in BMD to normal levels between T_3_ and T_4_, two remained with low BMD, and two were lost at the follow-up. 

From T_1_ to T_3_, the RCI revealed that 11/12 athletes (92%) did not increase their TBLH BMD *Z*-scores, and none increased their lumbar spine BMD *Z*-scores. From T_3_ to T_5_, none increased their TBLH BMD *Z*-scores, while 5/9 (56%) increased their lumbar spine BMD *Z*-scores. From T_1_ to T_5_, none increased their TBLH BMD *Z*-scores, while 5/9 (56%) increased lumbar spine BMD *Z*-scores. Four athletes exhibited stable femur neck BMC and BMD, while five exhibited increased femur neck BMC and BMD. Five athletes exhibited stable total hip BMC and BMD, while four athletes showed an increase in total hip BMC and BMD from T_1_ to T_5_ (Table 3).

**Table 2:** Fixed effects and random components of the linear mixed model.

	Fixed Effects Parameter Estimates				Random Components		
		Estimate	95% CI				
Variable		(SE)	Low–High	*p*-Value		Variance	ICC
Body composition							
FFM (kg)	Intercept	57.3 (1.7)	53.9–60.7		Intercept	38.5	
	Slope	1.1 (0.1)	0.8–1.4	<0.001	Slope	0.2	0.981
					Residual	0.8	
Bone health							
Lumbar BMD (g/cm^2^)	Intercept	1.048 (0.019)	1.039–1.098		Intercept	0.001	
	Slope	0.021 (0.008)	0.006–0.037	0.022	Slope	4.9 × 10^−4^	0.362
					Residual	0.002	
Lumbar BMC (g)	Intercept	62.0 (2.8)	55.6–67.4		Intercept	58.9	
	Slope	2.6 (0.9)	0.8–4.3	0.015	Slope	5.3	0.643
					Residual	32.7	
Lumbar *Z*-score	Intercept	−0.77 (0.17)	−1.10 to −0.44		Intercept	0.36	
	Slope	0.04 (0.02)	0.01–0.07	0.029	Slope	0.00	0.953
					Residual	0.02	
Femur neck BMD (g/cm^2^)	Intercept	1.093 (0.033)	1.029–1.157		Intercept	0.014	
	Slope	0.012 (0.002)	0.008–0.017	<0.001	Slope	4.4 × 10^−5^	0.984
					Residual	2.2 × 10^−4^	
Femur neck BMC (g)	Intercept	5.8 (0.3)	5.2–6.4		Intercept	1.21	
	Slope	0.1 (0.0)	0.07–0.13	<0.001	Slope	0.00	0.992
					Residual	0.01	
Total hip BMD (g/cm^2^)	Intercept	1.123 (0.032)	1.060–1.185		Intercept	0.013	
	Slope	0.005 (0.002)	0.001–0.008	0.029	Slope	3.2 × 10^−5^	0.993
					Residual	9.6 × 10^−5^	
Total hip BMC (g)	Intercept	40.0 (1.9)	36.3–43.6		Intercept	45.2	
	Slope	0.4 (0.1)	0.2–0.5	<0.001	Slope	0.05	0.996
					Residual	0.2	
TBLH BMD (g/cm^2^)	Intercept	1.157 (0.024)	1.109–1.204		Intercept	0.007	
	Slope	0.013 (0.003)	0.008–0.019	<0.001	Slope	7.2 × 10^−5^	0.986
					Residual	1.1 × 10^−4^	
TBLH BMC (g)	Intercept	2719 (131)	2461–2976		Intercept	2.23 × 10^5^	
	Slope	61 (9)	43–78	<0.001	Slope	902	0.997
					Residual	731	
Resting metabolic rate and energy availability							
RMR_ratio_	Intercept	1.03 (0.01)	1.01–1.05		Intercept	7.96 × 10^−4^	
	Slope	−0.01 (0.01)	−0.02 to −0.0	0.016	Slope	4.80 × 10^−6^	0.275
					Residual	0.00210	
EA (kcal/kg FFM/day)	Intercept	50.1 (3.6)	43.6–57.1		Intercept	122.1	
	Slope	−0.7 (0.9)	−2.6 to 1.2	0.475	Slope	3.0	0.653
					Residual	64.8	
Disordered eating behavior and exercise addiction							
EAI-Y	Intercept	17.9 (0.7)	16.7–19.2		Intercept	3.1	
	Slope	−0.7 (0.2)	−1.2 to −0.3	0.005	Slope	0.1	0.442
					Residual	3.9	
Performance							
VO_2peak_ (mL/kg/min)	Intercept	61.3 (1.3)	58.7–64.0		Intercept	16.7	
	Slope	2.8 (0.4)	2.1–3.5	<0.001	Slope	0.3	0.629
					Residual	9.9	
Force (Newton)	Intercept	2464 (106)	2257–2671		Intercept	108,766	
	Slope	91 (24)	45–137	<0.001	Slope	70	0.649
					Residual	58,885	
Power (watt)	Intercept	1115 (49)	1020–1210		Intercept	28,831	
	Slope	29 (6)	17–41	<0.001	Slope	146	0.912
					Residual	2797	

Number of observations per variable: 54; groups: ID 13. BEDA-Q: Brief Eating Disorder in Athletes Questionnaire; BMC: bone mineral content; BMD: bone mineral density; BMI: body mass index; CI: confidence interval; DLS: Drive for Leanness Scale; EA: energy availability; EAI-Y: Exercise Addiction Inventory for Youth; EEE: exercise energy expenditure; EI: energy intake; FFM: fat-free mass; ICC: intra-class correlation; RMR: resting metabolic rate; SE: standard error; TBLH: total body less head; VO_2peak_: peak oxygen uptake.

**Table 3:** Bone mass values during the 3-year period (*n* = 13).

	Descriptive					Reliable Change ^a^ Shown in Absolute Values with % in Brackets								
	T_1_	T_2_	T_3_	T_4_	T_5_	T_1_–T_3_ (*n* = 12)			T_3_–T_5_ (*n* = 9)			T_1_–T_5_ (*n* = 9)		
	(*n* = 13)	(*n* = 13)	(*n* = 12)	(*n* = 12)	(*n* = 9)	Dec	Stable	Inc	Dec	Stable	Inc	Dec	Stable	Inc
TBLH														
BMC (g)	2764 ± 459	2848 ± 438	2952 ± 422	2995 ± 422	3002 ± 323	0 (0%)	4 (33%)	8 (67%)	0 (0%)	8 (89%)	1 (11%)	0 (0%)	1 (11%)	8 (89%)
BMD (g/cm^2^)	1.164 ± 0.080	1.189 ± 0.076	1.205 ± 0.069	1.207 ± 0.066	1.215 ± 0.057	0 (0%)	4 (33%)	8 (67%)	0 (0%)	5 (56%)	4 (44%)	0 (0%)	1 (11%)	8 (89%)
*Z*-score	0.26 ± 0.84	0.32 ± 0.73	0.16 ± 0.72	0.19 ± 0.90	−0.05 ± 0.62	4 (33%)	7 (58%)	1 (8%)	4 (44%)	5 (56%)	0 (0%)	5 (56%)	4 (44%)	0 (0%)
L1–L4														
BMC (g)	64.1 ± 11.0	67.3 ± 12.2	71.1 ± 12.6	73.7 ± 10.7	75.0 ± 9.0	4 (33%)	1 (8%)	7 (58%)	0 (0%)	4 (44%)	5 (56%)	4 (44%)	0 (0%)	5 (56%)
BMD (g/cm^2^)	1.066 ± 0.083	1.093 ± 0.081	1.119 ± 0.087	1.139 ± 0.078	1.150 ± 0.069	5 (42%)	1 (8%)	6 (50%)	0 (0%)	4 (44%)	5 (56%)	4 (44%)	0 (0%)	5 (56%)
*Z*-score	−0.70 ± 0.61	−0.79 ± 0.57	−0.72 ± 0.62	−0.59 ± 0.57	−0.51 ± 0.51	4 (33%)	8 (67%)	0 (0%)	0 (0%)	4 (44%)	5 (56%)	1 (11%)	3 (33%)	5 (56%)
Femur Neck														
BMC (g)	5.9 ± 1.1	6.0 ± 1.1	6.2 ± 1.0	6.3 ± 1.1	6.2 ± 0.6	0 (0%)	9 (75%)	3 (25%)	0 (0%)	9 (100%)	0 (0%)	0 (0%)	4 (44%)	5 (56%)
BMD (g/cm^2^)	1.104 ± 0.119	1.117 ± 0.121	1.141 ± 0.110	1.153 ± 0.130	1.130 ± 0.088	0 (0%)	7 (58%)	5 (42%)	0 (0%)	7 (78%)	2 (22%)	0 (0%)	4 (44%)	5 (56%)
Total Hip														
BMC (g)	40.4 ± 6.6	40.7 ± 6.4	41.6 ± 6.1	41.9± 6.2	41.4 ± 3.6	0 (0%)	10 (83%)	2 (17%)	0 (0%)	9 (100%)	0 (0%)	0 (0%)	5 (56%)	4 (44%)
BMD (g/cm^2^)	1.129 ± 0.114	1.130 ± 0.110	1.142 ± 0.101	1.147 ± 0.109	1.131 ± 0.074	1 (8%)	10 (83%)	1 (8%)	0 (0%)	8 (89%)	1 (11%)	0 (0%)	5 (56%)	4 (44%)

Descriptive values are presented as mean ± standard deviation. ^a^ Decrease or increase indicates a critical value lower than −1.96 or higher than 1.96. BMC: bone mineral content; BMD: bone mineral density; Dec: decrease; Inc: increase; TBLH: total body less head.

## 4. Discussion

To the best of our knowledge, this is the first study monitoring REDs indicators in adolescent male athletes attending elite sports high schools using a multiple-year longitudinal design.

Our major finding was that although the athletes had an overall enhanced performance, more than one-third had poor bone health in the lumbar spine at baseline, and most of these athletes either lost or did not achieve the expected pubertal bone mineral accrual during the longitudinal period. 

### 4.1. Bone Health

Promoting physical exercise in combination with healthy eating habits during bone development is a key element for optimizing bone strength [10]. Men reach their highest bone mass at an age of ~20 years, with an accumulation of ~25% of total adult BMD between 13 and 19 years of age [10]. Furthermore, participation in high-impact loading sports is associated with increased BMD and BMC in the femur neck compared to low-impact repetitive sports such as running, cross-country skiing [29], and cycling [11]. However, poor bone health and osteoporosis is not caused merely by the loss of BMD during adulthood but may also result from a lack of optimal accumulated BMD during childhood and adolescence [10]. Hence, securing optimal bone health for adolescent athletes involved in low-impact sports is vital. It is worrying that 38% of the young athletes in the present study had low bone mass at baseline, during the peak window of optimal BMD accrual. Of these athletes, only one showed an increase in *Z*-score to above −1, two remained stable, and two were lost to follow-up. As such, these athletes might exhibit impaired bone health at the beginning of their senior athletic career, increasing the risk of bone stress injury and the potential early onset of osteopenia and osteoporosis, as reported previously [11,14]. In the present study, our model revealed an overall increase in TBLH BMD (~1%, ~0.013 g/cm^2^) and BMC (~2%, ~ 61 g), as well as in lumbar spine BMD (~2%, ~0.021 g/cm^2^), BMC (~4%, ~2.6 g), and *Z*-score (~5%, ~0.04) every 6 months. However, our exploratory RCI sub-analysis revealed a more troubling pattern. None of the athletes showed an increase in their lumbar spine *Z*-scores during T_1_–T_3_, and only ~50% of them showed improvements in lumbar spine *Z*-scores during T_3_–T_5,_ despite the fact that strength training was a regular part of the athletes’ training regimen throughout the three-year period. This is a concern, since athletes have some of the best opportunities to increase their bone strength during the investigated timeframe [10]. Furthermore, low BMD is a primary indicator when diagnosing REDs and should raise concerns and further scrutiny of athletes [30] since research in adults has found negative correlations between a high total training load and bone health [31]. Physical exercise in combination with sufficient nutritional status during bone development are key elements for good bone health [10]. Research has shown that jumping interventions can have positive effects on BMC in adolescent cyclists and swimmers [12,13], highlighting the importance of elite endurance sports high schools focusing on the risk of poor bone health and including sufficient nutrients. Weight training is another form of activity that can be implemented to facilitate increased bone health [32]. More knowledge needs to be implemented among athletes, coaches, and schools, including specific bone loading exercises within low-impact repetitive and non-weight-bearing endurance sports, to ensure that athletes have the best possible bone health when transitioning to adulthood, where bone accrual stops.

### 4.2. Energy Availability

Five cross-sectional studies have measured EA among male adolescents engaged in various sports [5,6,7,8,9]. Three studies found LEA (24–47%) defined as <30 kcal/kg FFM/day among athletes competing in climbing [5], cross-country running [6], and soccer [7]. In the present study, the mean EA at baseline was 50 ± 16 kcal/kg FFM/day, with no changes during the longitudinal period. We did identify EA <30 kcal/kg FFM/day in three athletes (23%) at different time points, similar to the incidence of 24–30% reported by others [6,7]. Measuring EA every six months may not, however, be suitable to identify LEA in young male athletes. A recent study by Jurov et al. [33] suggests that most of the negative effects of LEA in male athletes occur at a range of 9–25 kcal/kg FFM/day. Burke et al. [34] highlight that LEA is any mismatch between dietary EI and EEE that leaves the body’s total energy needs unmet, and while adaptable LEA is exposure to a reduction in EA that is associated with benign effects, problematic LEA is associated with REDs signs and/or symptoms and represents a maladaptive response. However, it is highly possible that the threshold for problematic LEA among adolescent males is higher compared to adults due to the energy requirements for growth, development, and puberty [35]. Hence, studies on adolescent athletes utilize different definitions of LEA, ranging from <30 kcal/kg FFM/day [5,6,7] to <45 kcal/kg FFM/day [8,9]. Characterizing adolescent athletes with LEA based on measurements of EA should be interpreted with caution, and more research on LEA in young athletes is necessary. 

### 4.3. Resting Metabolic Rate

RMR has been widely used as a surrogate marker for LEA in exercising adult females [36,37], and some evidence is starting to emerge in male athletes [35]. Reduced RMR has been reported in a male athlete involved in mixed martial arts during extreme weight reductions [38], in well-trained and elite male endurance athletes training excessively [39,40], and in male ballet dancers [37]. To the best of our knowledge, no studies have monitored RMR in adolescent male athletes from a multiple-year longitudinal perspective. Assessing RMR among adolescents can be problematic due to potentially biasing factors. Equations used to calculate predicted RMR are derived from an adult population [41], and the current cut-off value established for low RMR_ratio_ (<0.90) was originally established in adult women [42]. To the best of our knowledge, few studies have investigated RMR among adolescent athletes, and only among male soccer players [43,44,45]. In these studies, RMR_ratio_ seems to fluctuate between 0.84 and a maximum of 1.33 [44,45], which is similar to our findings. One of the key mechanisms influencing RMR is FFM, as this is one of the largest determinants, accounting for up to 70% of individual variations and considered the largest component of total energy expenditure [46]. Our athletes increased their FFM throughout the three-year period, and this could account for the high RMR_ratio_ observed. However, the threshold limits among adolescents are potentially higher compared to adults due to the energetic requirements of growth, development, and puberty [47]. Despite our results showing small indications of RMR and RMR_ratio_ reduction during the teenage years, while FFM increased, the cut-off for normal RMR for adult females (RMR_ratio_ > 0.90) is most likely not applicable to the male adolescent population, warranting further research [35,47].

### 4.4. Psychological Parameters

Being lean is an obvious advantage, as is competing in sports such as cross-country skiing and biathlon. However, a focus on leanness and a low body mass is also linked to DE behaviors [1]. In the present study, none of the athletes showed any signs of DE behaviors during the study period, with baseline BEDA-Q scores similar to reports among male students (4.0 ± 0.8) [23]. Furthermore, over the longitudinal period, all individual EAI-Y scores decreased, indicating that these young male athletes developed more unproblematic training behavior during the 3 years in this elite sport environment.

### 4.5. Performance

It is hypothesized that problematic LEA may affect performance indirectly through various processes such as reduced glycogen storage, reduced protein synthesis, increased risk of illness and injury, and psychological and mental impairments [1,3]. Few studies have attempted to identify the impact of LEA on sports performance in male athletes [33,39,40,48]. The findings of these studies include altered body composition, mood disturbances, and reduced aerobic and anaerobic performance after 4–6 weeks of intensified training without an increase in EI [39,40]. Keay et al. [48] found reduced testosterone levels, lower body fat, and impaired cycling performance in male cyclists with problematic LEA. In the present study, both endurance capacity and muscle strength improved significantly in combination with increased training load during the 3 years. The improved performance, increased FFM and height, and maintenance of EA levels among the athletes in our study might indicate that the athletes maintained satisfactory nutritional intake and recovery during the period.

### 4.6. Strengths and Limitations

The main strength of the present study is its longitudinal design lasting 3 years with a total of five measuring points, which has not been used previously to investigate REDs in adolescent male athletes. Furthermore, we used gold-standard methods and best-practice guidelines for measuring body composition, bone health, RMR, and performance variables, as well as valid instruments for measuring psychological parameters. Despite the small number of included participants, this study indicates that conducting a longitudinal study involving laboratory measures over three years is feasible, but we acknowledge that some limitations exist. The sample can be considered a convenience sample, and due to logistical and economic reasons, we had no control group. True prevalence rates cannot be calculated, and the results cannot be generalized. Dropouts could potentially be due to REDs complications. Unfortunately, due to ethical considerations, we were not allowed to perform any follow-up on the dropouts. This may bias our results, resulting in only “healthy” athletes continuing in our study. The semi-structured dietary interview used for assessing EI could be considered more unprecise compared to weighed food records; nevertheless, we cannot be certain that the calculated EA levels were representative. The method used was selected due to ethical considerations, since the elite sports high school leaders were worried that weighed food records could trigger DE behavior among these young athletes. Data on micronutrient distributions, including calcium and vitamin D intake and supplementation, would provide valuable insight into better understanding athletes bone health issues, and future research should incorporate such data. Furthermore, excluding injured athletes from participation may have led to a survivorship bias. Future research should include injured athletes in the analysis to gain a better understanding of REDs. Finally, due to economic reasons as well as within-subject hormone production and fluctuation over a longer period [49], we chose not to include blood sampling. This is warranted in further research, to get a better understanding of LEA and the effects on adolescent hormone concentrations. 

## 5. Conclusions

To the authors’ knowledge, this is the first study to assess REDs-related parameters in a multi-year longitudinal setting among male endurance athletes attending sport-specific elite high schools. Key findings were that overall performance improvements were observed along with the absence of problematic LEA, DE behavior, and exercise addiction during the period. However, 5 out of 13 athletes exhibited poor bone health in the lumbar spine at baseline, and most of the included athletes either lost or did not achieve the expected pubertal bone mineral accrual during the 3-year period. This is a major and important finding and highlights the importance of elite sports high schools focusing on the risk of poor bone health in male athletes and gaining more knowledge on specific bone loading exercises within low-impact repetitive and non-weight-bearing endurance sports to ensure that athletes have the best possible bone health when transitioning to adulthood. Monitoring bone health and including bone-accumulating exercises for young male athletes in endurance sports is recommended, and further research should include both endurance and non-endurance sports. 

## Figures and Tables

**Table 1:** Descriptive characteristics of the included athletes at baseline.

Variables	Athletes (*n* = 13)
Anthropometry	
Age (years)	16.3 ± 0.4
Height (cm)	179.4 ± 7.6
Weight (kg)	63.6 ± 8.2
BMI (kg/m^2^)	19.7 ± 1.6
FFM (kg) ^†^	57.4 ± 6.6
Body fat (%) ^†^	11.5 ± 3.3
Resting metabolic rate and energy availability	
RMR (kcal/kg/day)	31.9 ± 2.7
RMR_ratio_	1.03 ± 0.07
EA (kcal/kg FFM/day)	50.7 ± 15.8
Disordered eating behaviors and exercise addiction	
BEDA-Q	0.0 (0.0–0.0)
DLS	3.8 (3.2–4.7)
EAI-Y	18.0 (17.0–20.0)
Performance	
VO_2peak_ (mL/kg/minute)	61.5 ± 5.3
Force (Newton)	2754 ± 566
Power (Watt)	1167 ± 200
Training volume (h/year)	531 ± 36

Data are presented as mean ± standard deviation. Questionnaire data are presented as median (interquartile range 25–75). ^†^ measured using dual-energy X-ray absorptiometry. BEDA-Q: Brief Eating Disorder in Athletes Questionnaire; BMI: body mass index; DLS: Drive for Leanness Scale; EA: energy availability; EAI-Y: Exercise Addiction Inventory for Youth; FFM: fat-free mass; RMR: resting metabolic rate; VO_2peak_: peak oxygen uptake.

## Data Availability

Data sharing is unavailable due to ethical restrictions.
